# Abstract of the Proceedings of the Buffalo Medical Association

**Published:** 1856-08

**Authors:** Sanford B. Hunt


ART. III.—*Abstract of the Proceedings of the Buffalo Medical Association.*

TUESDAY EVENING, July 2, 1856.

The association met at its rooms.

Present—The President, Dr. Eastman, (in the chair); Drs. Hamilton, Burwell, Nichols, Hadley, Wyckoff, Newman, Howell, Root, Lemon, Strong, Treat, Miner, Baker, White, Hunt, Rochester and Boardman.

The minutes of the preceding meeting were read and approved.

Drs. E. L. Holmes and — Whitehead, were elected to membership on complying with the by-laws.

Prof. Geo. Hadley, previously appointed to report on the subject of Hair Dyes, made an interesting verbal report.

*Prof. Hadley* stated that he had been unable to find any treatises upon the subject which afforded him any assistance, and he was, therefore, obliged to speak from his own knowledge of the chemical constitution of nearly all the various articles in use: either as dyes, depilatories, or for improving its growth and appearance. To this he should principally confine himself.

The history of the use both of hair dyes, and of preparations for beautifying the hair, extends to the remotest antiquity, and no people, however degraded or however elevated in the scale of civilization, has ever existed, among whom we may not find their use to be prevalent.

At the present time, as probably in all others, the object sought by a hair dye is the production of a color darker than the one worn. We never hear of any article being used for decolorizing the hair, or substituting an auburn or light-brown for any darker hue.

All the various hair dyes in use are made from the different salts of two metals, viz: lead and silver. Other substances *may* be used, but they are either too expensive or too injurious to the hair itself.

1st, *Lead.* Several salts of lead are used; but the most common lead dye is that made from litharge and lime. Equal parts of lime and litharge are rubbed into a paste, with sufficient water to furnish the proper consistence. This paste is then very freely applied to the hair, being well rubbed in to it, and thickly laid on. It is kept moist by oil or india-rubber cloth, and allowed to remain for from three to eight hours—is usually put on at night and kept on during sleep. The color which results is unexceptionable, being a fine dark black.

It will be noticed that this dye is applied to the skin, but does not color it. Prof. H. stated that he had applied it to his arm for a period of six hours. The hairs beneath were died, but the skin remained colorless. This is the more curious, from the fact that, physiologically, the hair and the epidermis are the same in their elemental construction. There is, however, but little doubt that the coloring properties of lead depend on the deposition of a sulphuret in the hair. Most protein substances emit sulphuretted hydrogen on the action of caustic alkalis. In the process of "hulling corn," by placing it in ley, the grain is blackened by the alkali. The process of this hair dye, then, is as follows: The litharge is in part dissolved by the caustic lime, and the lead, thus rendered soluble, penetrates the hair and is deposited as a sulphuret of lead. It is remarkable that in the use of this dye the *dandruff* is colored.

The formulæ of lead dyes vary. Sometimes white lead is used; sometimes whiting or flour are added to prevent the too caustic action of the lime. Some formulæ, like the prescriptions of the older physicians, contain a dozen or more ingredients, only a few of which are essential.

Prof. Hadley stated that he had heard no complaints of the effect of this dye on the skin. The hair, however, is rendered brittle by its use, and must be oiled to prevent its breaking. In spite of any precaution the ends will crack off, and its use is often abandoned for this reason, though the color produced is unexceptionable.

"Twigg's Hair Dye" is simply a weak solution of acetate of lead to which precipitated sulphur is added. It is put up by almost every druggist, and sold under many different names. The continued use of it does, in many instances, cause the hair to grow darker, though Prof. H. had never seen a black color produced by it. It also checks the formation of dandruff. In its effects it corrugates the skin, which becomes harsh, and the person using it assumes a peculiar, old, wrinkled look. This, however, soon passes off. The directions, unlike other dyes, insist on its being well rubbed in.

*Silver Dyes.* These are much more used than the lead. The effects of light on the salts of silver are well known. The chloride of silver when exposed to light in a moist state, turns black; and the photographic process is entirely similar, except that the effect of the light upon the iodide or bromide of silver is not visible until subjected to reägents. In a similar manner these dyes are put up; some of them consisting simply of a single bottle of a solution of a salt of silver when the action of light is alone relied on as the reägent, ("dyes without preparation); or in other cases a reägent accompanies the package ("dyes with preparation.")

Nitrate of silver is the salt used in the manufacture of all these dyes, but it does not exist as a nitrate in the dye. *Pure* nitrate of silver is not affected by light; it may be exposed in a window for years, if tightly corked, without change. The change of color is uniformly due to the presence of organic matter, as when paper is wrapped around the sticks, or where the crystals come in contact with the cork of the bottle.

In dying the hair many use the "dye without preparation," and expose to light. This is a slow process, and the hair should be thoroughly cleansed by soap and soda before its application. In experimenting in the laboratory, Prof. H. found that by boiling the hair in caustic soda he was able to produce a deep permanent black with a dye of only one-eighth the ordinary strength. No dyes now sold contain simple nitrate of silver; all are more or less mixed.

In the use of dyes with preparation, a mordant accompanies the dye. In twenty or thirty different dyes which Prof. Hadley had analyzed, this preparation consisted uniformly of a weak solution of gallic acid, in the proportion of one grain of the acid to one hundred grains of water. This reduces the salt to an oxyde, instantaneously, constituting a large saving in time, while the tint is better than in the dyes without preparation. These latter leave the hair with a reddish tinge, particularly in reflected light, unless the dye be very strong, or twice used.

In some dyes three bottles are put up, one of which is only to be used on the failure of the other two. This third bottle always contains a little sulphuret of sodium, potassium, or ammonia. In two hair dyes manufactured in Philadelphia, no solution of gallic acid is put up, but instead of it the second bottle contains sulphuret of potassium or sodium. The objections to this plan are serious. The odor of sulphuretted hydrogen is offensive, it is very caustic, and acts on the skin, so that if not used with care the hair may be scraped off easily with the back of a knife, or pulled out by handsfull.

The nitrate of silver acts upon the hair as upon other organic tissues. It kills it, producing a firm dry, hard eschar, which, in the case of an ulcer, protects the parts beneath. All of these hair dyes are warranted not to stain the skin; but all of them will stain it if they come in contact.

The per centage of silver in the dyes sold varies very much. Prof. H had analyzed nearly all the dyes sold, and found the per centage to vary from four to eighteen per cent. of metallic silver. The followlng will illustrate: One hundred parts of dye contain

| | | | | |
|---|---|---|---|---|
| In Ballard's Hair Dye, - - | 6 | per cent. | of | silver. |
| " Batchelor's (Utica prep.) - - | 16 | " | " | " |
| " (New York prep.) - | 18 | " | " | " |
| " Phalon's, - - - - - | 18 | " | " | " |
| " Christadoro's, - - - | 15 | " | " | " |
| " Mathews, - - - - - | 5 | " | " | " |

(Many others were given.)

This per centage, however, furnishes no index to the actual value or cost of the dye, inasmuch as the size of the bottle varies, and also the price. Thus each bottle of these various dyes contains of solid silver:

| | | | | | |
|---|---|---|---|---|---|
| Ballard's Dye, - - | silver | to the | value of | 10 | cents. |
| Phalon's " - - - | " | " | " | 10 | " |
| Batchelor's (Utica prep.) | " | " | " | 8 | " |
| " (N. Y. prep.) | " | " | " | 8 | " |
| Christadoro's - - - | " | " | " | 8 | " |
| Clirehugh's - - . | " | " | " | 8 | " |

All these are sold at one dollar per bottle, leaving an enormous profit. Mathew's dye is sold at half a dollar per bottle, each bottle containing 7c. of metallic silver. The quantity of silver thus subtracted from the currency is very large. One firm in this city used last year (in a period of thirteen months) 1100 ounces of silver coin.

There is a common belief among the Chinese, that a certain weed, taken internally, would cause the hair to grow black, and attempts have been made by speculators here to discover it, and introduce it in this country.

Prof. Hadley continued his remarks, taking up briefly the subject of preparations for beautifying the hair. These promise everything: to cure baldness, prevent dandruff and falling of the hair, etc., etc. Oils and fats are mostly used in them. The great difficulty in the use of free oils is their liability to become rancid. A few years ago nearly all the liquid hair oils were made from lard oil, colored. The usual color was red, produced by alkanet, the red color of which is soluble in oils.

More recently castor oil is almost exclusively used. When pure it is too thick for convenient use, and is, therefore, mixed with strong alcohol, as follows:

℞ Castor oil, ℥x.

Alcohol, ℥ij.

Essential oils, ʒij.

These latter are used as perfumes.

The various Tricopheri, the Renovators, and Kathairons, are pretty nearly alike. All contain alcohol in large proportion, and all have tincture of cantharides as a stimulant. Various perfumes are used to convey a distinctive character. The following formula is a good one, and a fair sample of these articles:

℞ Castor oil, ℥vj.
Alcohol, ℥xxvj.
Tr. cantharides, ℥j.
Essential oils, ℥jss.

Colognes are simply perfumed alcohol.

*Depilatories* are usually powders, and almost without exception contain arsenic. The best of these is an old Turkish one, and consists of orpiment (ter sulphuret of arsenic) ground with quicklime and water into a paste, which is applied to the hair. The gland secreting the hair is not destroyed, and the hair is reproduced. Of course these arsenical preparations, (and all which Prof. H. had examined were arsenical) are injurious. They would be less so were real orpiment used; but the orpiment of commerce is almost pure white arsenic. The alkaline sulphurets may be used as depilatories, but are unpleasant on account of the odor of sulphuretted hydrogen.

*Prof. White* stated in reference to medicines used internally to color the hair, that nitrate of silver does not color the hair of persons whose skin is stained by its use. In one "blue man" he had seen, the hair resembled that of an albino. He inquired what cosmetic would be most efficient in removing the discolored spots on the skin often produced during pregnancy.

Prof. Hadley thought corrosive sublimate the best. He would mention that the "Balm of a Thousand Flowers," so largely sold, was simply a solution of soap highly perfumed.

*Prof. Hamilton* moved that "Prof. Hadley be requested to reduce his valuable report to writing and deposit with the secretary."* Carried.

*Prof. White* mentioned glycerine as a very valuable cosmetic.

*Prof. Hadley* stated that it would soon be largely used, and is very convenient, from its property of combining in any proportion with water or alcohol.

*Dr. Strong*, from the "Committee on Fistula in Ano," reported not prepared.

*Dr. Boardman*, from the "Committee on Foreign Bodies in the Alimentary Canal," reported not prepared.

*Prof. Hamilton* presented a case of "Lupus non exedens," with the accompanying history:

### *Lupus non exedens Serpiginosus.*

Michael Falin, æt. 38. Moderate drinker; a laborer; not scrophulous; generally healthy; about two years and a-half since a tubercle formd on the lower part of the sternum. It opened and ulcerated. Since then the disease has gradually spread until now it covers the front of the chest, being about eight inches in its perpendicular diameter, and about five in its transverse. The whole of this surface looks like the cicatrix of a burn, the skin being thrown into irregular folds, by narrow cord-like bands; it is also smooth, thin and vascular. All around the margins are heavy crusts like rupia. The disease seems to involve and disorganize the skin without the interposition of ulceration, or even of the formation of tubercles, so far as I can see. The whole surface is occasionally covered with furfuraceous scales. It is sometimes painful, but not at present.

He has now been using Fowler's sol. of arsenic, M. 15 ter die, during about two weeks. He is also living upon a milk and farinaceous diet. The improvement is very marked.

*Prof. Rochester* mentioned a case with which he had been associated. During one year the patient pursued a general tonic treatment of cod liver oil, the iodides, etc., without benefit. He then took for three months 5 drops of Fowler's solution *ter die*, when the disease became checked, and has not, for the last four years, progressed. The old cicatrices are still present.

*Dr. Wyckoff* presented a specimen of tænia solium, expelled by pumpkin-

---

* Circumstances attending the publication of these minutes, have induced the secretary to publish the foregoing abstract of his remarks.

seed emulsion. The patient was a man 60 years of age, who has occasionally expelled portions of the worm for five or six years.

Last fall Dr. W. gave him ol. terebinth in large doses, which brought away about thirteen feet of the worm. He had been subject to pain in the pit of the stomach and vertigo. June 38th Dr. W. prescribed the emulsion' of which the patient took more largely than directed. He bruised a quart of the seeds, and making a decoction, drank it all during the evening of the 28th and morning of the 29th. He followed this by castor oil, and passed on the 30th, six feet of the worm. Dr. W. had, as yet, no opportunity to examine it to see whether or no the head was expelled.

*Prof. Hamilton* then presented a specimen of

*Inorganic unadherent concretions in the Elbow-joint.*

The case of a man about 35 years old, a brakeman on the State Line R. R., whose arm was amputated on account of a severe compound injury. There are four small unorganized concretions in the joint: the smallest being of the size of a lentil, and the largest of the size of a bean. The largest is situated at the fore and upper part of the trochlea of the humerus, at which point there is an oval fossa into which the concretion was received, but from which it must have been displaced upward underneath the elongated capsule of the joint whenever the forearm was forcibly flexed upon the arm, since the coronoid process of the ulna falls now into the same fossa.

The three smaller concretions were situated in the olecranon fossa, within the capsule also; but they were not reached by the olecranon process in forced extension of the arm.

The patient says he never experienced any difficulty in this joint.

The concretions are of a pearly white color, and irregularly oval in form.

*Prof. White* recalled the case of diabetes mellitus mentioned at a previous meeting, to inquire if Prof. Hadley could state the weight of sugar obtained from the urine of a single day. He would state that the patient had been under the care of Dr. Mitchell, of Brooklyn, who had changed the alkaline treatment for carbonate of ammonia, in small doses, and a purely animal diet. This had been attempted before, but failed. She bore the diet better this time, and was somewhat improved.

*Prof. Hadley* stated that the sp. gr. of the urine was 1040. The secretion for a single day amounted to eleven pints, and the amount of well-drained crystalized sugar obtained had not been weighed, but nearly filled a pint bottle.

Dr. Daniel Devening was proposed as a candidate for membership, by Dr. Newman.

*Dr. Wyckoff* inquired if the resolution offered by Prof. White at the preceding meeting, adopting the report of the chairman of the committee on pneumonia, was in order.

*The Secretary* then read the resolution.

*Dr. Burwell* suggested that as the continuation of the chairman's report (that on the Diagnostic value of the Buffy Coat) was not yet published, and many members would desire to read it before the discussion, it would be better to allow the resolution to lie over another month.

*Prof. White* concurred in the reasons of Dr. Burwell, and accordingly the resolution was laid on the table till the August meeting.

And the association then adjourned.

SANFORD B. HUNT, Secretary.

